# GenX exposure induces neurodevelopmental impairment and synaptic toxicity in hESC-derived cerebral organoids

**DOI:** 10.1016/j.mtbio.2026.103458

**Published:** 2026-07-15

**Authors:** Sung-Ae Hyun, Young-Ju Lee, Moon Yi Ko, Euijun Min, Heejin Park, Younhee Kim, Dae Youn Hwang, Byoung-Seok Lee, Minhan Ka

**Affiliations:** a Center for Convergence Toxicology Research, Korea Institute of Toxicology, Daejeon, 34114, Republic of Korea; b Center for Regulatory Toxicology Research, Korea Institute of Toxicology, Daejeon, 34114, Republic of Korea; c Department of Biomaterials Science, College of Natural Resources and Life Science/Life and Industry Convergence Research Institute, Pusan National University, Miryang, 50463, Republic of Korea; d Department of Biochemistry, Chungnam National University, Daejeon, 34134, Republic of Korea; e Center for Toxicologic Pathology Research, Korea Institute of Toxicology, Daejeon, 34114, Republic of Korea; f Human and Environmental Toxicology, University of Science and Technology, Daejeon, 34114, Republic of Korea

**Keywords:** GenX, Neurodevelopment, Neurogenesis, Gliogenesis, Dendritic spines, Synapse

## Abstract

GenX, also known as hexafluoropropylene oxide dimer acid (HFPO-DA), was introduced in 2009 as a purportedly safer alternative to perfluorooctanoic acid (PFOA). However, nearly two decades of use have raised increasing safety concerns owing to its reported association with multiple forms of organ damage. However, only a few studies have systematically evaluated the molecular mechanisms underlying the neurotoxicity of GenX exposure. In this study, we aimed to investigate the neurodevelopmental impairments and synaptic toxicity induced by GenX during cortical development using human embryonic stem cell-derived cerebral organoids. We found that early exposure to GenX resulted in reduced organoid size and concentration-dependent deficits in basal neural progenitor cell proliferation. Moreover, GenX exposure prior to differentiation impaired synapse formation and decreased the number of excitatory synapses. We also found that early GenX exposure disrupted neural activity and altered neural network organization. RNA sequencing and Gene Ontology analyses revealed that these effects were associated with dysregulation of neuropeptide signaling pathways, synapse assembly, and postsynaptic organization. Collectively, these impairments in neurogenesis and neural activity indicate that GenX exerts neurotoxic effects and may pose a potential risk factor for neurodevelopmental disorders.

## 1 Introduction

Per- and polyfluoroalkyl substances (PFASs) are a class of synthetic organic chemicals widely used across industries for their high durability, water resistance, and non-stick properties [1,2]. Owing to these characteristics, PFASs have been detected globally, including in remote regions such as the Arctic and Antarctic [3]. Exposure to PFASs has been linked to various adverse health outcomes, including certain cancers [4,5], endocrine system disruption [6], and neurological impairments [7,8]. Among these compounds, perfluorooctanoic acid (PFOA) has been extensively studied, with research demonstrating associations with multiple health conditions, including metabolic disorders [9], congenital anomalies [10], and neurodevelopmental conditions [11,12]. These findings have heightened public health concern regarding PFAS use and prompted the development of alternative compounds [13]. One such alternative, hexafluoropropylene oxide dimer acid (HFPO-DA; marketed as GenX), was introduced in 2009 as a purportedly safer replacement for PFOA (EPA Report, 2021), although its toxicological profile remains insufficiently characterized.

GenX has been detected in multiple environmental matrices, including surface water, groundwater, and atmospheric emissions [[14], [15], [16]]. Although the primary exposure routes to GenX have not been fully elucidated, substantial concentrations have been identified in critical drinking water resources [17,18]. GenX exhibits limited bioaccumulation in human tissues, which may be attributed to its relatively recent commercial introduction and enhanced biodegradation characteristics [19,20]. However, its structural similarity to PFOA suggests comparable environmental persistence, while its shorter carbon chain structure may facilitate greater environmental mobility, thereby posing potential long-term ecological challenges (EPA Report, 2021). Animal studies have demonstrated that GenX exposure can affect organs such as the liver, pancreas, and kidneys [21,22], with prenatal exposure potentially causing metabolic disorders and liver damage in offspring, indicating transgenerational risks [23]. In zebrafish, GenX exposure impairs developmental processes, induces oxidative stress, and elicits neurotoxic effects [24]. Furthermore, exposure prior to differentiation induces persistent nuclear and mitochondrial alterations, disrupting key neuronal markers, and may increase the risk of neurodegenerative disease [25].

The development of the brain—more than any other organ—relies on the precise regulation of neural progenitor self-renewal and differentiation to establish functional neural networks [26]. In particular, the cerebral cortex, a critical region responsible for complex behaviors [27], depends on tightly regulated neural progenitor proliferation and maintenance of appropriate size and function [28]. Disruptions in neural progenitor function and cortical development can impair neural circuitry, which has been associated with a range of neurodevelopmental disorders, including cognitive impairment, autism spectrum disorder (ASD), attention-deficit/hyperactivity disorder (ADHD), and seizure disorders [29]. In the present study, we employed human embryonic stem cell (hESC)-derived cerebral organoids generated in our laboratory to investigate the impact of GenX exposure on cortical development and synaptic activity in the developing cerebral cortex. Morphological alterations, cell proliferation, and apoptosis induced by GenX were systematically evaluated in hESC-derived cerebral organoids. We found that GenX impaired neural activity and synaptic transmission in these organoids. Transcriptomics analyses were performed to elucidate the mechanisms underlying GenX-induced neurodevelopmental toxicity. Collectively, our findings provide insights into the pathological processes that contribute to neurodevelopmental conditions associated with GenX exposure.

## 2 Materials and methods

### 2.1 Chemicals

Hexafluoropropylene oxide dimer acid (HFPO-DA, also known as GenX; CAS No. 13252-13-6) was purchased from Sigma-Aldrich and dissolved in dimethyl sulfoxide (DMSO) to prepare a 100 mM stock solution. For all experiments, final working concentrations of 10 μM and 100 μM were applied.

### 2.2 Ethics statement

All experiments involving human embryonic stem cells (hESCs) was approved by the Public Institutional Bioethics Committee (approval numbers: P01-202106-41-001) designated by the Ministry of Health and Welfare. The methods were conducted in accordance with approved guidelines.

### 2.3 hESC culture and cerebral organoids differentiation

The H9 hESC line was obtained from the WiCell Research Institute (Madison, WI, USA). H9 hESCs were cultured in TeSR™-Plus medium (STEMCELL Technologies, #100-1130) on Matrigel hESC-Qualified Matrix (Corning, #354277)-coated plates. When cells reached 80–90% confluence, they were rinsed with Dulbecco's phosphate-buffered saline (DPBS) and detached using ReLeSR™ solution (STEMCELL Technologies, #100-0483) at 37°C for 3–4 min. After removing the ReLeSR™ solution, cells were rinsed with TeSR™-Plus medium and reseeded at a 1:5 ratio in TeSR™-Plus medium supplemented with 10 μM ROCK inhibitor Y-27632 (STEMCELL Technologies, #72307) for the first 24 h. Cells were maintained with daily medium changes and incubated at 37°C in a 5% CO_2_ incubator.

Cerebral organoids were generated according to the manufacturer's protocol (STEMdiff™ Cerebral Organoid Kit, STEMCELL Technologies, #08570). Briefly, hESCs were seeded into 96-well plates using embryoid body (EB) seeding medium and cultured in EB formation medium for five days. The newly formed EBs were then transferred to 24-well plates and cultured in induction medium. After 48 h, EBs with translucent edges were embedded in Matrigel and transferred to 6-well plates containing expansion medium. Three days later, EBs that developed budding neuroepithelia were identified as newly formed organoids. These organoids were transferred to an Ultra-Low Adherent ClinoReactor (CELVIVO, #10006-12) containing maturation medium (STEMCELL Technologies, #08571) and maintained in a ClinoStar stress-free 3D incubator (CELVIVO, Model 30003) at 37°C in 5% CO_2_. Culture medium was completely replaced every 3–4 days.

### 2.4 TUNEL staining

Apoptotic cell death in day 20 cerebral organoids was assessed using the DeadEnd™ Fluorometric TUNEL System (Promega, G3250) according to the manufacturer's instructions. Briefly, organoid sections were fixed in 4% formaldehyde in PBS, permeabilized with 0.5% Triton X-100 in PBS, and re-fixed prior to equilibration and labeling with TdT reaction mix for 60 min at 37°C in the dark. Nuclei were counterstained with DAPI using Vectashield mounting medium. TUNEL-positive cells were detected by fluorescence microscopy and quantified as a percentage of total DAPI-positive nuclei per section from four independent organoid samples per experimental group.

### 2.5 Real-time polymerase chain reaction (RT-PCR)

RT-qPCR was performed following our previously described method [30]. Briefly, total RNA was isolated from cerebral organoids using TRIzol reagent (Thermo Fisher Scientific, #15596018). For cDNA synthesis, 1 μg of total RNA was reverse transcribed using the GoScript™ Reverse Transcription Mix (Promega, #A2791). qPCR was performed using GoTaq qPCR Master Mix (Promega, #A6102) on a CFX Connect Real-Time PCR Detection System (Bio-Rad) under the following conditions: 95°C for 3 min, followed by 40 cycles of 95°C for 10 s and 56°C for 30 s. Data were quantified using CFX Maestro software (Bio-Rad) and analyzed using the 2^(-ΔΔCt) method with GAPDH as the housekeeping gene for normalization. Primer sequences are listed in Table 1.

**Table 1:** List of primer sequences for qRT-PCR.

Gene	Forward primer	Reverse primer
OCT4	CCTGAAGCAGAAGAGGATCACC	AAAGCGGCAGATGGTCGTTTGG
KLF4	AAAGAGTTCCCATCTCAAGGC	GTAGTGCCTGGTCAGTTCATC
PAX6	CAACTCCATCAGTTCCAACG	TGGATAATGGGTTCTCTCAAACTCT
Nestin	TCCAGAAACTCAAGCACCA	AAATTCTCCAGGTTCCATGC
MAP2	CTGCTTTACAGGGTAGCACAA	TTGAGTATGGCAAACGGTCTG
NeuN	TACGCAGCCTACAGATACGCTC	TGGTTCCAATGCTGTAGGTCGC
TBR1	GTCACCGCCTACCAGAACAC	ACAGCCGGTGTAGATCGTG
CTIP2	CTCCCTTTGGATGCCAGTGTCA	GGCTCCAGGTAGATGCGGAAG
GFAP	AGGTCCATGTGGAGCTTGAC	GCCATTGCCTCATACTGCGT
S100β	GAAGAAATCCGAACTGAAGGAGC	TCCTGGAAGTCACATTCGCCGT
DSCAML1	CCACCATCAGCCACATGAACG	GACAGGCACCTCTTACGTTTAT
WNT7A	AGGAGAAGGCTCACAAATGGGC	CGGCAATGATGGCGTAGGTGAA
SEMA4A	GTCAGCCTTGGCCTCTTATTAT	CCAACTCCATCCTGCACTATC
FABP7	CATCAGGACTCTCAGCACATT	TCCAGGCTAACAACAGACTTAC
GAPDH	GGTGAAGCAGGCGTCGGAGG	GAGGGCAATGCCAGCCCCAG

### 2.6 Immunoblotting

Immunoblotting was performed following our previously described method [31]. Cerebral organoids were lysed using RIPA Lysis and Extraction Buffer (Thermo Fisher Scientific, 89901), and protein concentrations were measured using the Pierce™ BCA Protein Assay Kit (Thermo Fisher Scientific, 23225). Proteins were resolved on 8%, 10%, or 15% SDS-PAGE gels and transferred onto PVDF membranes (Thermo Fisher Scientific). The membranes were incubated overnight at 4°C with primary antibodies listed in Table 2. Afterward, the membranes were washed five times with PBS and incubated with secondary antibodies (Thermo Fisher Scientific, 65-6120 and 62-6520) for 1 h at room temperature. Following another five PBS washes, immunodetection was performed using the ECL reagent (Thermo Fisher Scientific, 34075). Band intensities were analyzed using ImageJ software from three independent experiments. β-actin served as an internal loading control for normalization.

**Table 2:** Antibodies for immunoblotting and immunostaining.

Antibodies	Conc.	Company	Catalog no.
Mouse anti-SOX2	1:1000	Abcam	ab79351
Rabbit anti-TBR2	1:200	Abcam	ab23345
Mouse anti-MAP2	1:1000	Thermo	13-1500
Rabbit anti-TBR1	1:1000	Abcam	ab31940
Mouse anti-NeuN	1:1000	Abcam	ab104224
Rabbit anti-GFAP	1:1000	Neuroimics	RA22101
Rat anti-Ki-67	1:1000	Invitrogen	11-5698-82
Rat anti-CTIP2	1:1000	Abcam	ab18465
Guinea pig anti-VGLUT	1:1000	synaptic system	135304
Mouse anti-VGAT	1:1000	synaptic system	131011
Mouse anti-Synaptophysin	1:1000	Santa Cruz	sc17750
Mouse anti-PSD95	1:1000	Santa Cruz	sc32290
Mouse anti-GAD67	1:1000	synaptic system	198211
Rat anti-Gephyrin	1:1000	synaptic system	147208
Mouse anti-β-actin	1:1000	Therom	A5316

### 2.7 Immunostaining

Immunostaining of cerebral organoids was performed as described previously [32]. Cerebral organoids were collected and fixed in 4% PFA at 4°C overnight. After fixation, they were washed twice with PBS and dehydrated in 30% sucrose solution at 4°C for 48 h. The organoids were then embedded in optimal cutting temperature (OCT) compound and frozen at −80°C. Cryosections were prepared at 20 μm thickness using a cryostat (Leica, CM1950). The sections were air-dried, washed with PBS, and blocked, followed by overnight incubation at 4°C with primary antibodies as listed in Table 2. The sections were washed five times with PBS, followed by a 1-h incubation at room temperature with Alexa Fluor-conjugated secondary antibodies (Thermo Fisher Scientific). Nuclei were stained with DAPI for 10 min, and the sections were mounted using Fluoromount-G™ Mounting Medium (Thermo Fisher Scientific, 00-4958-02). Immunofluorescence imaging was performed using an Olympus FV3000 fluorescence microscope with Olympus software (Olympus Life Sciences). The experiment was independently repeated three times under identical conditions, with four wells allocated for each concentration.

### 2.8 Multi-electrode array (MEA) system

Neural activity and network dynamics were measured according to previously described protocols [33] with minor modifications. Cerebral organoids cultured for 40 days were transferred onto poly-D-lysine/laminin-coated MEA plates (Axion Biosystems, M768-tMEA-48W) and maintained in maturation medium. Following a 24-h stabilization period, spontaneous neuronal activity was recorded for 15 min per organoid at a sampling rate of 12.5 kHz at 37°C under a humidified atmosphere containing 5% CO_2_ using the Maestro MEA system (Axion Biosystems). Raw signals were bandpass filtered at 200–3000 Hz. Data acquisition and analysis were performed using Axion Integrated Studio (AxIS) software version 2.5.2 and the Neural Metric Tool (Axion Biosystems).

Spikes were detected using an adaptive threshold crossing algorithm with the detection threshold set at ±6 times the standard deviation (SD) of baseline noise. Electrodes exhibiting a minimum firing rate of ≥5 spikes/min during the recording period were classified as active electrodes. Single-electrode bursts were identified using an inter-spike interval (ISI)-based algorithm and defined as a minimum of 5 spikes occurring within a maximum ISI of 100 ms. Network bursts were defined as synchronized burst events consisting of at least 50 spikes involving ≥35% of participating electrodes within a maximum ISI of 100 ms. Synchrony analyses were performed using a 20-ms window size. Number of active electrodes, weighted mean firing rate, burst frequency, and burst duration were extracted for quantitative analysis.

### 2.9 Library preparation and sequencing

For control and test RNAs, the construction of library was performed using QuantSeq 3′ mRNA-Seq Library Prep Kit (Lexogen, Inc.) according to the manufacturer's instructions. In brief, each 500 ng total RNA were prepared and an oligo-dT primer containing an Illumina-compatible sequence at its 5′ end was hybridized to the RNA and reverse transcription was performed. After degradation of the RNA template, second strand synthesis was initiated by a random primer containing an Illumina-compatible linker sequence at its 5′ end. The double-stranded library was purified by using magnetic beads to remove all reaction components. The library was amplified to add the complete adapter sequences required for cluster generation. The finished library is purified from PCR components. High-throughput sequencing was performed as single-end 75 sequencing using NextSeq 500 (Illumina, Inc.).

### 2.10 Data analysis

QuantSeq 3’ mRNA-Seq reads were aligned using Bowtie2 [34]. Bowtie2 indices were either generated from genome assembly sequence or the representative transcript sequences for aligning to the genome and transcriptome. The alignment file was used for assembling transcripts, estimating their abundances and detecting differential expression of genes. Differentially expressed gene were determined based on counts from unique and multiple alignments using coverage in Bedtools [35]. The RC (Read Count) data were processed based on quantile normalization method using EdgeR within R (R development Core Team, 2016) using Bioconductor [36]. Gene classification was based on searches done by DAVID (http://david.abcc.ncifcrf.gov/) and Medline databases (http://www.ncbi.nlm.nih.gov/).

### 2.11 Statistical analysis

Normal distribution was tested by the Kolmogorov–Smirnov test, and variance was compared. Unless otherwise stated, statistical significance was determined by two-tailed student t-test for two sample comparisons or by one-way or two-way analysis of variance (ANOVA) followed by the Bonferroni post hoc test for multiple comparisons. Data were analyzed using GraphPad Prism (GraphPad Software, Inc.) and presented as mean (±) SEM. ∗*p* < 0.05, ∗∗*p* < 0.01, ∗∗∗*p* < 0.001. P values were indicated in Figure legends.

## 3 Results

### 3.1 Generation and characterization of hESC-derived cerebral organoids

To investigate the neurodevelopmental impairments caused by GenX exposure, we generated hESC-derived cerebral organoids following the differentiation and exposure protocols outlined in Fig. 1A. We first optimized morphological progression at each stage, including embryoid body (EB) formation, neuroectoderm development, neuroepithelial expansion, and cerebral organoid generation (Fig. 1A). To evaluate morphogenesis, we performed immunohistochemical staining for cell type–specific markers in the cerebral organoids on day 40. SOX2-positive neural progenitor cells are localized along the ventricular zone (VZ), whereas TBR2-positive intermediate progenitors are located in the adjacent subventricular zone (SVZ). Mature neurons, marked by MAP2 and NeuN, were distributed throughout the organoid, with enrichment in the outer regions. TBR1-positive deep-layer cortical neurons were located superficial to the VZ, demonstrating appropriate inside-out cortical layer formation. Additionally, GFAP-positive astrocytes were distributed within the organoids, indicating the emergence of glial cells (Fig. 1B). These results confirm that the cerebral organoids successfully recapitulate key features of human cortical development. Next, we analyzed cell type–specific marker expression in day 40 cerebral organoids using qRT-PCR. The pluripotency markers OCT4 and KLF4 were highly expressed in undifferentiated hESCs but were nearly undetectable by day 40, confirming the loss of stemness during neural differentiation (Fig. 1C). In contrast, neural progenitor cell (NPC) markers PAX6 and Nestin were significantly upregulated in these organoids compared to those in undifferentiated hESCs (Fig. 1D). Neuronal markers MAP2 and NeuN also showed robust expression in the organoids, indicating successful neuronal differentiation (Fig. 1E). Moreover, cortical layer–specific markers TBR1 and CTIP2 were significantly upregulated in the organoids, demonstrating the acquisition of cortical identity (Fig. 1F). Furthermore, astrocytic markers GFAP and S100β were elevated in these organoids, indicating the emergence of glial populations (Fig. 1G). These results confirm that the cerebral organoids successfully recapitulate cortical development and contain NPCs, mature neurons, and astrocytes.

**Fig. 1:**
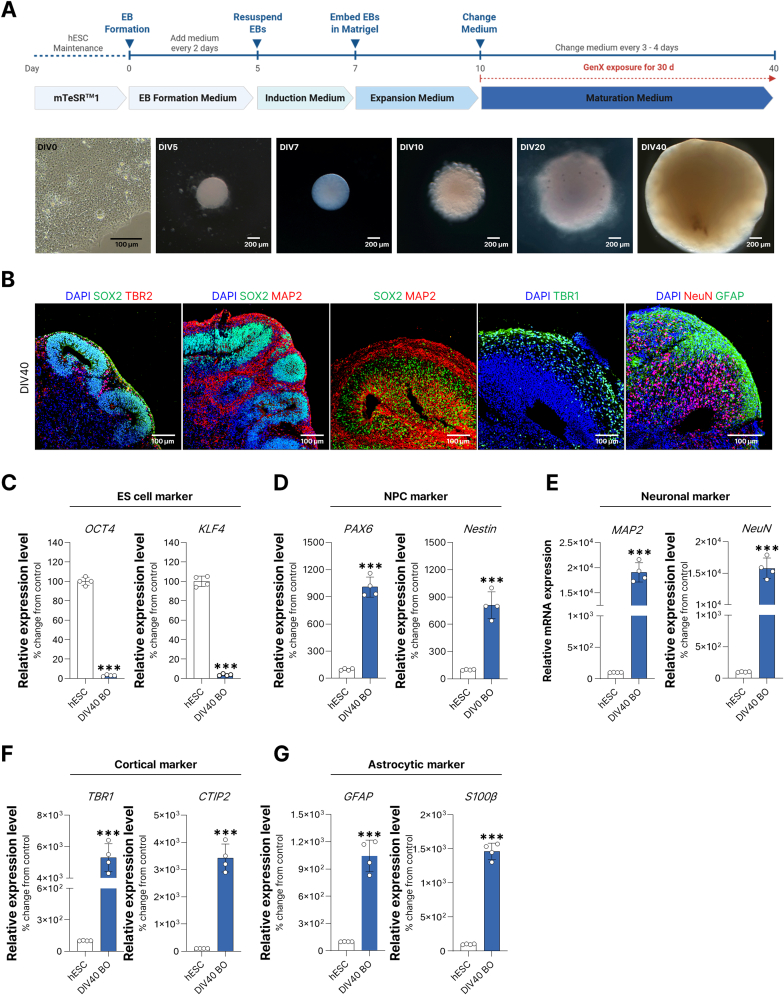
Generation and characterization of hESC-derived cerebral organoids for GenX exposure study (A) Schematic illustration of the differentiation process from human induced pluripotent stem cells (hESCs) to cerebral organoids, indicating the time point of GenX treatment. Representative bright-field images at each differentiation stage are shown below. (B) Immunohistochemical analysis of day 40 cerebral organoids stained for SOX2 (neural progenitor marker), TBR2 (intermediate progenitor marker), MAP2 (neuronal marker), TBR1 (cortical neuron marker), NeuN (neuronal marker), and GFAP (astrocytic marker). Scale bars, 100 μm. (C) Relative mRNA expression levels of pluripotency markers, (D) neural progenitor cell (NPC) markers, (E) neuronal markers, (F) cortical markers, and (G) astrocytic markers in hESCs and day 40 organoids. Data are presented as mean ± SEM (n = 4 independent experiments). Statistical significance was determined by one-way ANOVA followed by Dunnett's post-hoc test for multiple comparisons against the vehicle control group. ∗∗∗p < 0.001 vs. hESCs.

### 3.2 GenX exposure impairs NPC proliferation and reduces cerebral organoid size

To investigate the impact of GenX exposure on neurodevelopmental impairments, we examined the morphology of cerebral organoids exposed to GenX from day 10 to day 40, corresponding to the transition from the expansion phase to the onset of the maturation phase (Fig. 1A). In the control group, organoids formed prominent neuroepithelial loops on day 20 that persisted through day 30, with overall organoid size consistently increasing over time (Fig. 2A). In contrast, GenX-exposed organoids displayed significantly smaller sizes and reduced areas in a concentration-dependent manner compared with those in the control group (Fig. 2A). At 10 and 100 μM GenX, organoid sizes decreased by 23.0% and 34.4%, respectively, compared to unexposed control organoids at day 20; by day 30, their sizes reduced by 18.0% and 35.0%, respectively (Fig. 2A and B). To determine whether the observed reduction in organoid size was attributable to increased apoptotic cell death, TUNEL staining was performed at day 30. No significant difference in the number of TUNEL-positive cells was observed between vehicle control and GenX-exposed organoids (Supplementary Fig. S1), indicating that the size reduction reflects growth inhibition rather than apoptosis-driven cell loss. These results suggest that GenX exposure disrupts brain morphogenesis during cerebral organoid development.

**Fig. 2:**
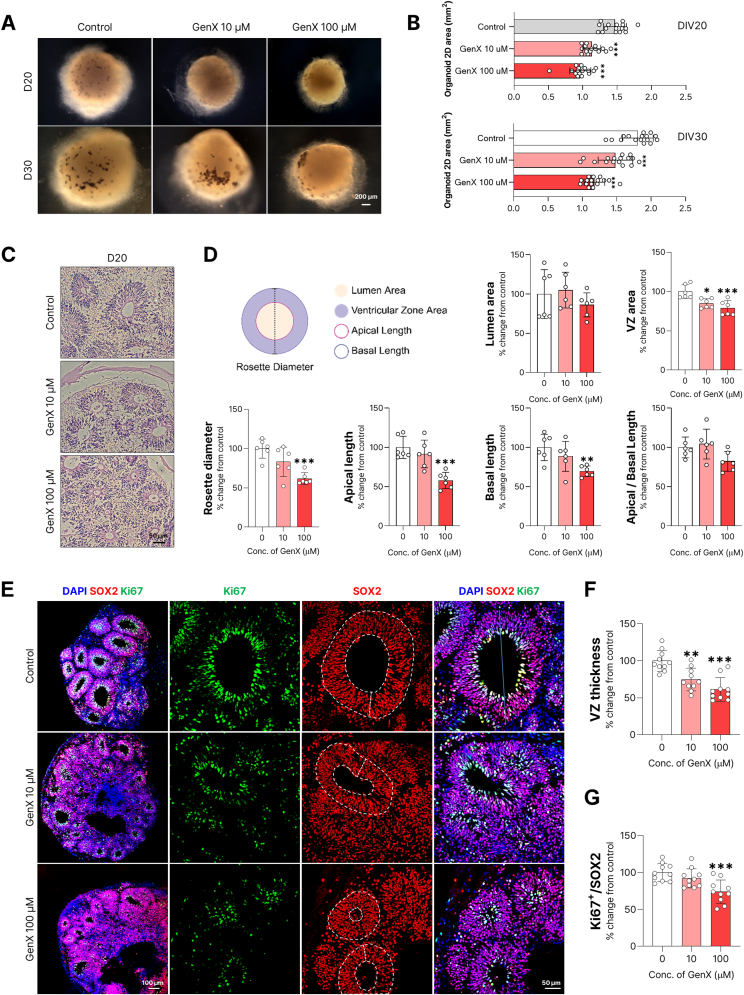
GenX exposure reduces the size and alters the structural features of cerebral organoids. (A) Representative bright-field images of cerebral organoids treated with control medium, 10 μM GenX, or 100 μM GenX at day 20 (upper panel) and day 30 (lower panel) of differentiation. Scale bars, 200 μm. (B) Quantification of organoid size at day 20 (upper) and day 30 (lower). Data are presented as mean ± SEM (n = 16). (C) Hematoxylin and eosin (H&E) staining of organoids at day 20. Scale bars, 50 μm. (D) Quantitative analysis of morphological parameters including lumen area, ventricular zone (VZ) area, rosette diameter, apical length, basal length, and apical/basal length ratio. Measurements were performed using ImageJ software with manual tracing. Data are presented as mean ± SEM (n = 6). (E) Immunohistochemical staining of day 20 organoids for Ki67 (green) and SOX2 (red). Nuclei were counterstained with DAPI (blue). Scale bars, 50 μm. (F) Quantification of VZ thickness based on SOX2^+^ area. Data are presented as mean ± SEM (n = 10). (G) Quantification of proliferative progenitors (Ki67^+^/SOX2^+^ double-positive cells). Data are presented as mean ± SEM (n = 10). Statistical significance was determined by one-way ANOVA followed by Dunnett's post-hoc test for multiple comparisons against the vehicle control group. ∗p < 0.05, ∗∗p < 0.01, ∗∗∗p < 0.001 vs. control.

To further investigate cortical structure, we examined the morphology of the organoids on day 20 by measuring the lumen area, VZ area, rosette diameter, and apical and basal length (Fig. 2C and D). On day 20, the lumen area was not significantly altered by GenX exposure at either 10 or 100 μM. However, treatment with 100 μM GenX reduced the VZ area by 21.0% compared to that in control organoids. Rosette diameter was significantly decreased by 38.3% in organoids exposed to 100 μM GenX. Similarly, apical and basal lengths were reduced by 42.1% and 30.4%, respectively, at this concentration. However, the apical-to-basal length ratio remained unchanged at both GenX concentrations (Fig. 2D). These findings suggest that GenX impairs NPC proliferation and cortical tissue expansion while preserving the polarized organization of the neuroepithelium. For day 30 organoids, VZ area was also significantly reduced in a concentration-dependent manner following GenX exposure (Supplementary Fig. S2).

Next, to investigate the impact of GenX on NPC proliferation, we performed immunostaining for SOX2 and Ki-67 in control and GenX-exposed organoids at day 20 (Fig. 2E). VZ thickness, measured by the SOX2^+^ area, was reduced by approximately 25.3% and 38.7% in organoids exposed to 10 μM and 100 μM GenX, respectively (Fig. 2F). Furthermore, the proportion of actively proliferating NPCs, identified as SOX2^+^/Ki-67^+^ double-positive cells, decreased by approximately 7.8% and 26.0% under 10 μM and 100 μM GenX exposure, respectively (Fig. 2G). These results indicate that GenX exposure suppresses NPC proliferation in a concentration-dependent manner, consistent with the observed reduction in VZ area and overall organoid size. The decrease in proliferative activity likely contributes to the impaired cortical expansion observed in GenX-treated organoids.

### 3.3 GenX exposure disrupts neuronal distribution and cortical marker expression in cerebral organoids

Next, to investigate the effects of GenX on cortical cell differentiation, we analyzed cell type–specific marker expression in organoids at day 40. The mRNA levels of NPC markers *PAX6* and *Nestin* were significantly downregulated in a concentration-dependent manner following GenX exposure (Fig. 3A). Consistently, the expression of neuronal markers *MAP2* and *NeuN* was also downregulated in a concentration-dependent manner in GenX-exposed organoids compared to that in the control organoids (Fig. 3B). Similarly, the cortical layer markers TBR1 and CTIP2 showed a concentration-dependent decrease following GenX exposure (Fig. 3C). Moreover, astrocytic markers GFAP and S100β were reduced in GenX-exposed organoids (Fig. 3D). These findings suggest that GenX impairs the differentiation and maturation of NPCs into neurons and astrocytes. The concentration-dependent reduction in both pan-neuronal and cortical layer–specific markers indicate disrupted cortical neurogenesis, potentially leading to altered cellular composition within the developing organoids.

**Fig. 3:**
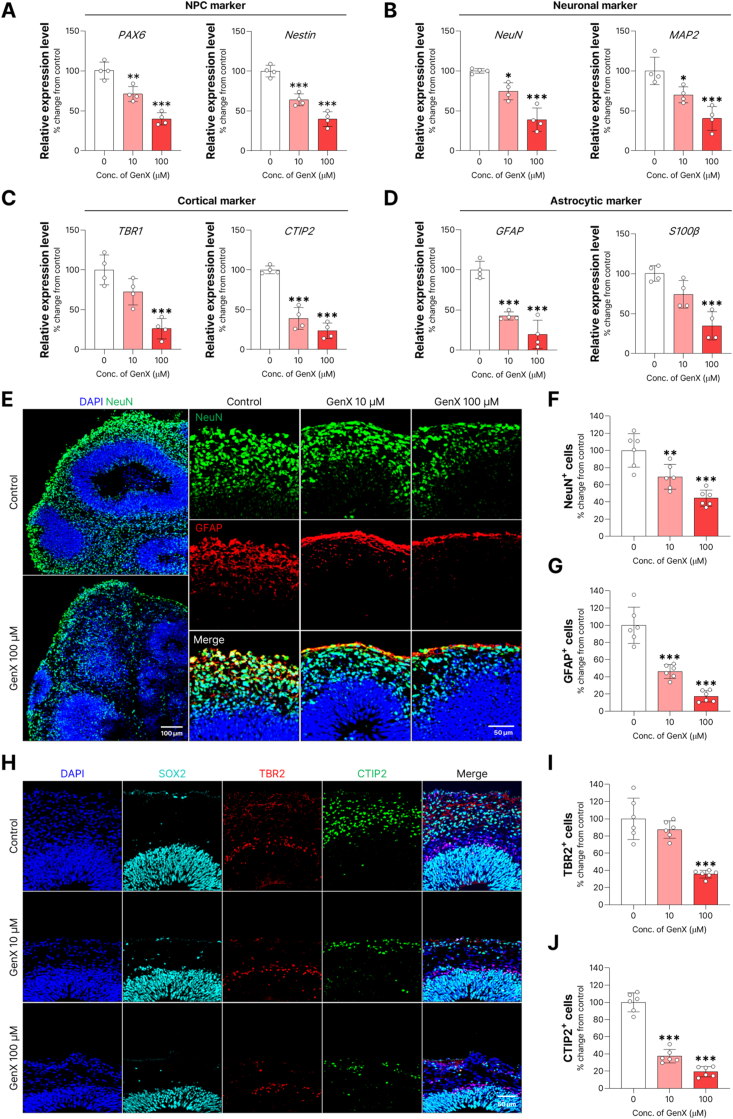
GenX exposure disrupts cellular composition and differentiation in cerebral organoids. (A) Relative mRNA expression levels of neural progenitor cell (NPC) markers (PAX6, Nestin), (B) neuronal markers (NeuN, MAP2), (C) cortical markers (TBR1, CTIP2), and (D) astrocytic markers (GFAP, S100β) in day 40 cerebral organoids exposed to control medium, 10 μM GenX, or 100 μM GenX. Data are presented as mean ± SEM (n = 4). (E) Immunohistochemical staining of day 40 cerebral organoids for NeuN (green) and GFAP (red). Nuclei were counterstained with DAPI (blue). Scale bars, 50 μm. (F) Quantification of NeuN^+^ cells. Data are presented as mean ± SEM (n = 6). (G) Quantification of GFAP^+^ cells. Data are presented as mean ± SEM (n = 6). (H) Immunohistochemical staining of day 40 cerebral organoids for SOX2 (magenta), TBR2 (red), and CTIP2 (green). Nuclei were counterstained with DAPI (blue). Scale bars, 50 μm. (I) Quantification of TBR2^+^ cells. Data are presented as mean ± SEM (n = 6). (J) Quantification of CTIP2^+^ cells. Data are presented as mean ± SEM (n = 6). Statistical significance was determined by one-way ANOVA followed by Dunnett's post-hoc test for multiple comparisons against the vehicle control group. ∗p < 0.05, ∗∗p < 0.01, ∗∗∗p < 0.001 vs. control.

To further characterize changes in cellular composition, we performed immunostaining for NeuN and GFAP at day 40 to quantify neurons and astrocytes, respectively. The number of NeuN^+^ cells was reduced by 30.7% and 55.6% in organoids exposed to 10 μM and 100 μM GenX, respectively, compared with that in the controls (Fig. 3E and F). Similarly, the number of GFAP^+^ cells decreased by 53.8% and 82.7% following exposure to 10 μM and 100 μM GenX, respectively (Fig. 3E and G). Next, we examined cortical progenitor and neuronal subpopulations via immunostaining for TBR2 and CTIP2. The number of TBR2^+^ intermediate progenitor cells was reduced by 12.5% and 64.4% in organoids exposed to 10 μM and 100 μM GenX, respectively (Fig. 3H and I). CTIP2^+^ deep-layer cortical neurons showed more pronounced reductions of 62.5% and 80.7% following exposure to 10 μM and 100 μM GenX, respectively (Fig. 3H and J). Collectively, these results demonstrate that GenX disrupts both neurogenesis and astrogenesis in a concentration-dependent manner, leading to decreased generation of cortical neurons and glial cells during cerebral organoid development.

### 3.4 GenX exposure disrupts synaptogenesis and neuronal activity in cerebral organoids

**Fig. 4:**
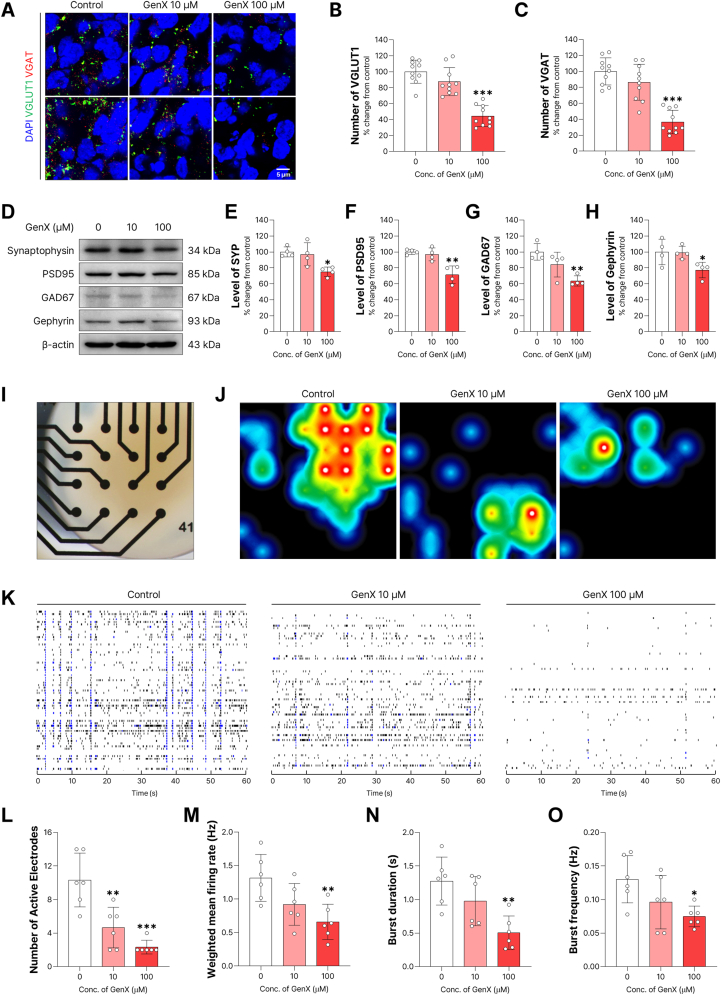
Abnormal synapse formation and function in neurons derived from GenX-exposed cerebral organoids. (A) Immunohistochemical staining of day 40 cerebral organoids for synaptic markers VGLUT1 (green, excitatory) and VGAT (red, inhibitory). Nuclei were counterstained with DAPI (blue). Scale bars, 5 μm. (B–C) Quantification of synaptic puncta density for (B) VGLUT1 and (C) VGAT. Data are presented as mean ± SEM (n = 10). (D) Immunoblot analysis of synaptic proteins including Synaptophysin, PSD95, GAD67, and Gephyrin in day 40 organoids. β-actin served as a loading control. (E–H) Quantification of protein expression levels normalized to β-actin for (E) Synaptophysin, (F) PSD95, (G) GAD67, and (H) Gephyrin. Data are presented as mean ± SEM (n = 4). (I) Representative image of cerebral organoids cultured on a multielectrode array (MEA) plate. (J) Representative neuronal activity heatmaps and (K) spike raster plots showing spontaneous neuronal activity recorded from day 40 organoids treated with control, 10 μM GenX, or 100 μM GenX. (L–O) Quantification of electrophysiological parameters including (L) number of active electrodes, (M) weighted mean firing rate, (N) burst duration, and (O) burst frequency. Data are presented as mean ± SEM (n = 6). Statistical significance was determined by one-way ANOVA followed by Dunnett's post-hoc test for multiple comparisons against the vehicle control group. ∗p < 0.05, ∗∗p < 0.01, ∗∗∗p < 0.001 vs. control.

To examine the effects of GenX on synaptogenesis, we performed immunostaining for VGLUT1 and VGAT, which label excitatory and inhibitory synapses, respectively. The density of VGLUT1 puncta was reduced by 12.4% and 55.5% in organoids exposed to 10 μM and 100 μM GenX, respectively, compared to that in controls (Fig. 4A and B). Similarly, VGAT puncta density was reduced in a concentration-dependent manner in GenX-exposed organoids (Fig. 4A and C).

To evaluate synaptic protein expression, we performed immunoblot analyses of pre- and postsynaptic markers in day 40 organoids. The excitatory presynaptic marker synaptophysin was downregulated by 3.0% and 24.9% following 10 μM and 100 μM GenX exposure, respectively (Fig. 4D and E). Similarly, the excitatory postsynaptic marker PSD95 was reduced by 3.1% and 28.7% in 10 μM and 100 μM GenX-exposed organoids, respectively (Fig. 4D and F). The inhibitory presynaptic marker GAD67 was reduced by 15.9% and 36.4% following 10 μM and 100 μM GenX exposure, respectively (Fig. 4D and G). Likewise, the inhibitory postsynaptic marker gephyrin was downregulated by 0.8% and 22.8% in 10 μM and 100 μM GenX-exposed organoids, respectively (Fig. 4D and H). This coordinated reduction in both pre- and postsynaptic markers suggests impaired synaptogenesis, which is consistent with the observed decrease in neuronal populations.

To evaluate the functional consequences of GenX exposure, we measured neural activity and network connectivity using a multielectrode array system. Organoids were cultured on MEA plates for 24 h prior to recordings (Fig. 4I). The validity of the recorded signals was confirmed by characteristic biphasic spike waveforms and pharmacological suppression with TTX (Supplementary Fig. S3). GenX-exposed organoids exhibited reduced neural activity and impaired network connectivity compared to those in controls, as illustrated by representative activity heatmaps and spike raster plots (Fig. 4J and K). The number of active electrodes, weighted mean firing rate, burst frequency, and burst duration all decreased in a concentration-dependent manner following GenX exposure (Fig. 4L–O). These electrophysiological findings indicate that GenX-induced reductions in neuronal populations and synaptic protein expression impair functional network activity, disrupting both spontaneous neuronal activity and synchronized network behavior in developing cerebral organoids.

### 3.5 GenX exposure alters transcriptomic profiles in cerebral organoids

To elucidate the molecular mechanism underlying GenX-induced cortical alterations, we performed QuantSeq 3′ mRNA sequencing on day 40 organoids treated with control medium, 10 μM GenX, or 100 μM GenX. Differentially expressed genes were identified based on a fold change >2.0 and a q-value <0.05.

**Fig. 5:**
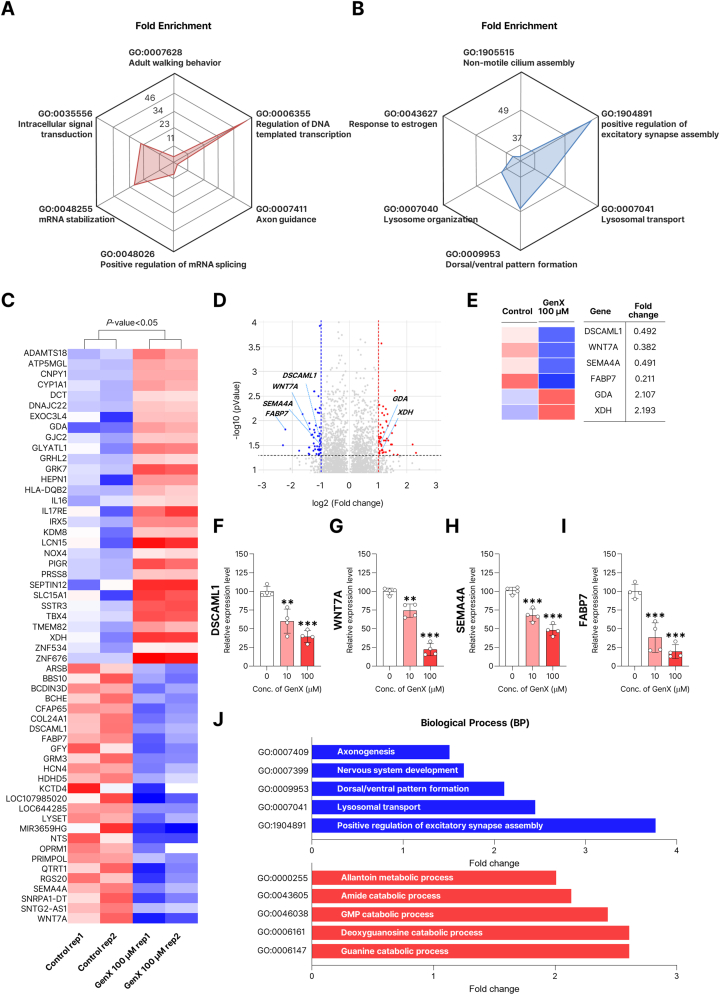
Transcriptomic analysis reveals GenX-induced dysregulation of neurodevelopmental genes. RNA from control and GenX-treated day 40 cerebral organoids was subjected to QuantSeq 3′ mRNA sequencing on a NextSeq 500/550 platform. Differentially expressed genes (DEGs) were identified based on fold change >2.0 and q-value <0.05. (A–B) Gene ontology (GO) enrichment analysis of biological processes in organoids treated with 10 μM GenX, showing (A) upregulated and (B) downregulated pathways. (C) Heatmap of DEGs in organoids treated with 100 μM GenX compared to control. (D) Volcano plot displaying upregulated (red) and downregulated (blue) DEGs (fold change >2.0, q-value <0.05). (E) Representative genes most significantly altered by 100 μM GenX treatment. (F–I) Bar graphs showing the relative mRNA expression levels of DSCAML1 (F), WNT7A (G), SEMA4A (H), and FABP7 (I) in human cerebral organoids exposed to vehicle control, 10 μM GenX, or 100 μM GenX. Expression levels were normalized to GAPDH and are presented as mean ± SEM from four independent organoid batches. Statistical significance was determined by one-way ANOVA followed by Dunnett's post-hoc test for multiple comparisons against the vehicle control group. ∗∗p < 0.01, ∗∗∗p < 0.001 versus vehicle control. (J) Gene ontology (GO) enrichment analysis of biological processes associated with 100 μM GenX treatment, showing downregulated (top) and upregulated (bottom) pathways.

Following 10 μM GenX exposure, Gene Ontology (GO) analysis revealed widespread transcriptional changes affecting critical neurodevelopmental processes. Upregulated genes were enriched in biological pathways related to regulation of DNA-templated transcription, axon guidance, positive regulation of mRNA splicing, intracellular signal transduction, and mRNA stabilization (Fig. 5A). In contrast, downregulated genes were associated with non-motile cilium assembly, excitatory synapse formation, lysosomal transport and organization, dorsal–ventral patterning, and estrogen response, suggesting impairment of neuronal connectivity, morphogenetic patterning, and intracellular degradation pathways (Fig. 5B).

Exposure to 100 μM GenX induced more pronounced transcriptomic alterations. Key neurodevelopmental regulators, including DSCAML1, WNT7A, SEMA4A, and FABP7, were downregulated, indicating disrupted axonal guidance, synaptic assembly, and NPC maintenance (Fig. 5C–E). These transcriptomic findings were independently validated by RT-qPCR at differentiation day 40, confirming concentration-dependent reductions in all four transcripts (Fig. 5F–I). Conversely, GDA and XDH were upregulated, suggesting activation of purine catabolic pathways and oxidative stress responses. Consistent with this, GO enrichment analysis revealed that the upregulated genes were significantly involved in guanine-, GMP-, and deoxyguanosine-catabolic processes (Fig. 5J). Collectively, these transcriptomic analyses demonstrate that GenX disrupts fundamental neurodevelopmental programs while promoting metabolic reprogramming in cerebral organoids.

## 4 Discussion

**Fig. 6:**
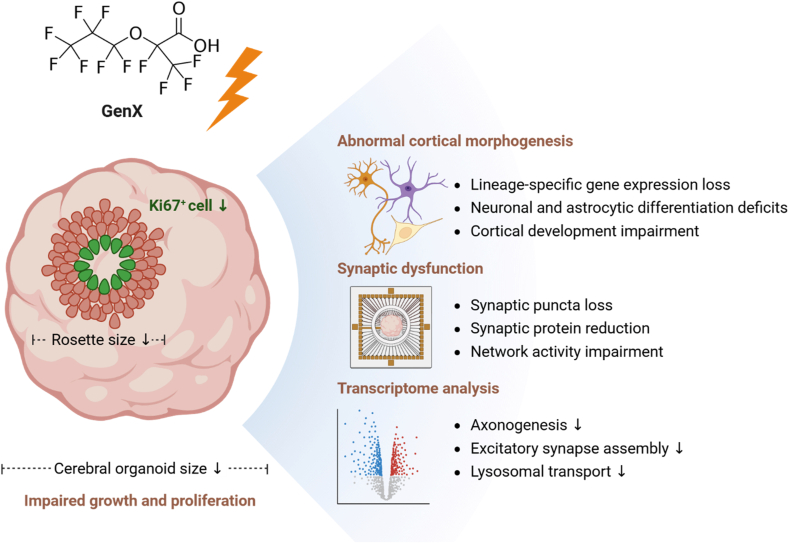
Schematic summary of GenX-induced neurodevelopmental alterations in human cerebral organoids. GenX exposure reduced organoid growth and neural progenitor proliferation, impaired cortical layer differentiation, and disrupted synaptic formation and neuronal activity. Transcriptomic analysis revealed downregulation of genes involved in axonogenesis, excitatory synapse assembly. These findings demonstrate that GenX disrupts critical neurodevelopmental processes including cortical morphogenesis and neuronal network formation.

Recent evidence indicates that GenX, a substitute for PFOA, has been detected globally in river water and may be more toxic than legacy PFAS. However, its potential neurodevelopmental effects following prenatal exposure remain poorly characterized. In this study, we demonstrated that GenX impairs brain development and synaptic function in hESC-derived cerebral organoids (Fig. 6). Our findings provide novel insights into the pathological mechanisms underlying GenX-associated neurodevelopmental disorders.

GenX has been detected in environmental water samples and treated drinking water, with half-life measurements indicating high stability [12]. GenX accumulates in biological tissues in a manner similar to other PFAS [37]. Early childhood exposure to PFAS, especially at low to moderate concentrations, has been significantly correlated with elevated ADHD symptoms in school-aged children [12]. PFAS alternatives have been shown to cause behavioral and neurodevelopmental effects in zebrafish. Early-life exposure to GenX altered genes related to neuronal differentiation and growth [24]. Embryonic exposure to various PFAS alternatives, including GenX, has been reported to induce alterations in larval locomotor and optomotor responses [[38], [39], [40]]. In rodents, early GenX exposure significantly alters placental thyroid hormone levels, potentially disrupting neurodevelopmental processes in offspring [41,42]. Consistent with these findings, we also observed that GenX impairs brain development in cerebral organoids. Collectively, these results suggest that PFAS exposure may increase the risk of neurodevelopmental disorders.

Environmental monitoring studies have reported GenX (HFPO-DA) and other PFAS at generally low concentration ranges (ng/L–μg/L) in various matrices [19,43,44]. In drinking water, one of the primary exposure sources for the general population, GenX concentrations typically range from 0.001 to 0.008 ng/mL (1–8 pg/mL), though levels as high as 0.63–0.8 ng/mL have been reported in more contaminated areas [14,18]. Biomonitoring studies have further confirmed human exposure to GenX, with HFPO-DA detected in urine (detection frequency ∼1.2%; concentration range 0.07–0.3 μg/L) [45] and in human serum at approximately 0.04–1 ng/mL [46], collectively supporting the relevance of GenX exposure under real-world environmental conditions. Notwithstanding these environmental concentration ranges, the working concentrations of 10 μM and 100 μM GenX employed in the present study were selected with reference to previous PFAS in vitro studies, encompassing a range from sub-cytotoxic to relatively high exposure conditions [47,48]. The application of elevated concentrations in cerebral organoid-based assays is often necessary to elicit measurable neurodevelopmental responses, given the absence of in vivo pharmacokinetic processes that govern tissue-level accumulation. Moreover, the distinct toxicokinetic properties of PFAS — including high protein binding affinity and considerable bioaccumulation potential — further support the scientific rationale for the experimental concentration range selected in this study [48,49].

Brain development occurs through a complex, sequential process that involves the proliferation and differentiation of NPCs, followed by neuronal migration and the establishment of neural circuits—all of which are critical for constructing a fully functional brain [[50], [51], [52]]. Brain size has been significantly correlated with neurodevelopmental conditions [53]. Changes in brain size—whether reduced (microcephaly) or enlarged (macrocephaly)—often serve as defining characteristics of various neurodevelopmental disorders [54]. Prenatal exposure to PFAS has been associated with neurodevelopmental conditions, such as ADHD and ASD [55,56]. These disorders can affect brain development and function, potentially leading to alterations in brain structure and size, although such changes are not directly measured in most studies [57,58]. In this study, we found that cerebral organoid size was significantly reduced after 30 days of GenX exposure. Correspondingly, the thickness of the VZ/SVZ in neural rosettes decreased in GenX-exposed cerebral organoids. Furthermore, administration of PFOA at doses exceeding 1 mg/kg resulted in fewer BrdU-labeled cells compared with controls [59]. Primary microcephaly is primarily caused by decreased proliferation of NPCs, resulting in a markedly reduced brain size while typically preserving overall brain architecture and cortical organization, although it is accompanied by intellectual disability [60]. Neurodevelopmental disorders are strongly influenced by both the quantity and functionality of cortical neurons. These conditions arise from disruptions during brain development and frequently involve abnormalities in neuronal number, migration, differentiation, and connectivity patterns, which in turn impair cognitive and behavioral functions [29,61]. In this study, we demonstrated that GenX exposure disrupts neurogenesis and astrogenesis in developing cerebral organoids.

The transcriptomic analysis and RT-qPCR-validated downregulation of FABP7, WNT7A, SEMA4A, and DSCAML1 provide a mechanistic framework linking GenX-induced transcriptomic alterations to the observed neurodevelopmental phenotypes. Suppression of *FABP7* corresponds to the reduced SOX2^+^ and TBR2^+^ progenitor populations [62], while decreased *WNT7A* expression implicates Wnt/β-catenin signaling disruption in the impaired cortical lamination [63]. Reduced *SEMA4A* and *DSCAML1* levels are consistent with the deficits in dendritic arborization, synaptic density, and MEA-recorded network activity [64,65], collectively indicating that GenX impairs progenitor maintenance, neuronal differentiation, and synaptogenesis in a convergent manner. In addition, GenX exposure further upregulated *GDA* and *XDH*, two genes implicated in purine catabolic pathways. Notably, the inhibition of de novo purine synthesis in the embryonic neocortex has previously been reported to markedly compromise brain development, manifesting as proliferative defects in neural stem and progenitor cells alongside delayed migration of immature neurons [66]. Furthermore, purinergic signaling is known to be indispensable for the maintenance of neural progenitor cells and the regulation of neuronal migration in the neocortical subventricular zone (SVZ) throughout brain development [67,68]. Taken together, our findings reveal that GenX exposure impairs both neurogenesis and astrogenesis in developing cerebral organoids.

Our transcriptomic analysis provides mechanistic clues linking GenX exposure to disruption of specific neurodevelopmental signaling pathways. WNT7A, which was significantly downregulated in a concentration-dependent manner, is a critical regulator of NPC proliferation, neuronal differentiation, and synaptogenesis through the canonical Wnt/β-catenin pathway [69,70]. Its downregulation is consistent with the reduced organoid growth and impaired neuronal differentiation observed in this study. Similarly, DSCAML1 and SEMA4A play essential roles in cortical neuron migration, dendritic arborization, and synaptic connectivity [71,72], and their reduced expression may contribute to the disrupted cortical organization and diminished electrophysiological activity detected by MEA. Furthermore, upregulation of XDH, which catalyzes purine catabolism and generates reactive oxygen species through its interconversion to xanthine oxidase [73], together with enrichment of purine catabolic pathways, suggests that oxidative stress may serve as an additional downstream effector of GenX-induced neurotoxicity [74]. These findings collectively suggest a potential dual mechanism of GenX-induced neurodevelopmental toxicity involving direct disruption of neurodevelopmental signaling and indirect cellular damage through oxidative stress. Future studies employing pathway-specific pharmacological inhibitors and genetic approaches will be required to establish the causal involvement of these pathways.

Synaptic dysfunction, characterized by structural and functional disruptions at synapses, is a critical factor in the pathogenesis of neurodevelopmental disorders [75,76]. Conditions such as ASD, ADHD, and intellectual disability are frequently associated with impaired synaptic development and function [77,78]. Synaptic genes, which regulate neuronal development and maintain the excitation–inhibition balance, are commonly implicated in these disorders when mutations occur [76]. In this study, we found that GenX exposure disrupted synaptic protein expression in developing cerebral organoids. Furthermore, GenX exposure downregulated genes related to axonogenesis and synapse assembly in these organoids. These genes were enriched in Gene Ontology (GO) terms such as axonogenesis (GO:0007409), nervous system development (GO:0007399), and positive regulation of excitatory synapse assembly (GO:1904891).

Spontaneous firing rate and network bursting activity are established hallmarks of cortical circuit maturation in vitro [79,80]. The reduction in the number of active electrodes in GenX-exposed organoids reflects a diminished capacity for network-level recruitment, consistent with the reduced neuronal populations observed by immunofluorescence. The decrease in weighted mean firing rate indicates suppressed excitatory neuronal output, while the concurrent reductions in burst frequency and burst duration suggest impaired network synchronization, indicative of disrupted excitatory-inhibitory balance and insufficient synaptic connectivity. During early cortical development, synchronized burst firing is essential for activity-dependent synaptic refinement and stabilization of nascent neural circuits [[81], [82], [83]]. These functional deficits are further supported by the RT-qPCR-confirmed downregulation of DSCAML1 and SEMA4A, known regulators of synaptic specificity and dendritic morphogenesis, respectively.

Consistent with our findings, the expression levels of BDNF, PSD95, and Syn proteins in the cerebral cortex and hippocampus of mice exposed to 8 mg/kg PFOS for 45 days were reduced [84]. Notably, exposure to PFAS induces substantial alterations in synaptic transmission by adversely affecting neurotransmitter systems and fundamental cellular mechanisms [85]. PFOS exposure has also been shown to impair brain development and directly affect neuronal network activity [85]. Exposure to a PFAS mixture disrupts cognitive function, learning, and memory in mice [86]. Epidemiological and meta-analyses further suggest that prenatal PFAS exposure is associated with neurodevelopmental disorders in children, including deficits in performance, intelligence, executive function, psychomotor skills, attention, and language [87,88].

Several inherent constraints of the cerebral organoid model must be considered when interpreting the present findings. Since cerebral organoids are derived exclusively from neuroectodermal progenitors, they lack a functional vascular network, blood-brain barrier (BBB), and innate immune cells (microglia). The absence of vascularization impairs nutrient and oxygen diffusion, leading to hypoxia and necrotic core formation, while the lack of the BBB prevents physiologically accurate compound exposure calibration. Furthermore, the absence of microglia—which originate from the yolk sac mesoderm and are central to neuroinflammation and neurotoxicity responses—means that immune-mediated components of toxicity cannot be captured in this system. Collectively, these structural limitations preclude direct extrapolation of the in vitro toxicity thresholds identified here to in vivo neurodevelopmental risk. Future work incorporating vascularized assembloids, BBB-on-chip platforms, or microglia-integrated neuroimmune organoids with physiologically based pharmacokinetic (PBPK) modeling will be essential to improve translational validity [89,90]. Additionally, although GFAP immunofluorescence was used to assess astroglial differentiation in this study, GFAP is also expressed in radial glial progenitors within the ventricular zone of cerebral organoids. While the concurrent downregulation of both GFAP and S100β at the transcriptional level supports impaired astrogenesis, co-immunostaining of GFAP with a mature astrocyte-specific marker such as S100β would enable more precise discrimination between radial glial progenitors and committed astrocytes. Incorporating such dual-labeling approaches in future investigations will be necessary to further delineate the astrocyte-specific effects of GenX exposure during cortical development.

In conclusion, GenX, primarily used as a substitute for PFOA, exerts detrimental effects on neural development similar to those observed with other PFASs, particularly PFOA. Usin cerebral organoid models, our findings demonstrate that GenX exposure reduces neuronal populations and disrupts synaptic formation and function during crucial stages of development. Transcriptomic analysis further identified dysregulation of multiple biological processes relevant to neurodevelopment, including axonogenesis, synapse assembly, neuropeptide signaling, and lysosomal function-related pathways, providing a hypothesis-generating framework for subsequent mechanistic investigation. However, experimental validation of these pathways was not performed in the present study, as the primary objective was to establish a cerebral organoid-based platform for characterizing the neurodevelopmental toxicity of GenX and to provide comprehensive phenotypic evidence across multiple domains of brain development. Dedicated follow-up studies will be necessary to systematically validate these candidate mechanistic pathways and to fully elucidate the molecular basis of GenX-induced neurodevelopmental injury.

## CRediT authorship contribution statement

**Sung-Ae Hyun:** Data curation, Formal analysis, Investigation, Methodology, Validation, Visualization, Writing – original draft. **Young-Ju Lee:** Data curation, Formal analysis, Investigation, Validation, Visualization. **Moon Yi Ko:** Data curation, Formal analysis, Investigation, Validation, Visualization. **Euijun Min:** Formal analysis, Visualization. **Heejin Park:** Data curation, Formal analysis. **Younhee Kim:** Formal analysis, Visualization. **Dae Youn Hwang:** Supervision, Writing – review & editing. **Byoung-Seok Lee:** Conceptualization, Data curation, Investigation, Project administration, Supervision, Validation, Writing – review & editing. **Minhan Ka:** Conceptualization, Data curation, Formal analysis, Investigation, Project administration, Supervision, Validation, Writing – original draft, Writing – review & editing.

## Declaration of competing interest

The authors declare that they have no known competing financial interests or personal relationships that could have appeared to influence the work reported in this paper.

## Figures and Tables

**figs1:**
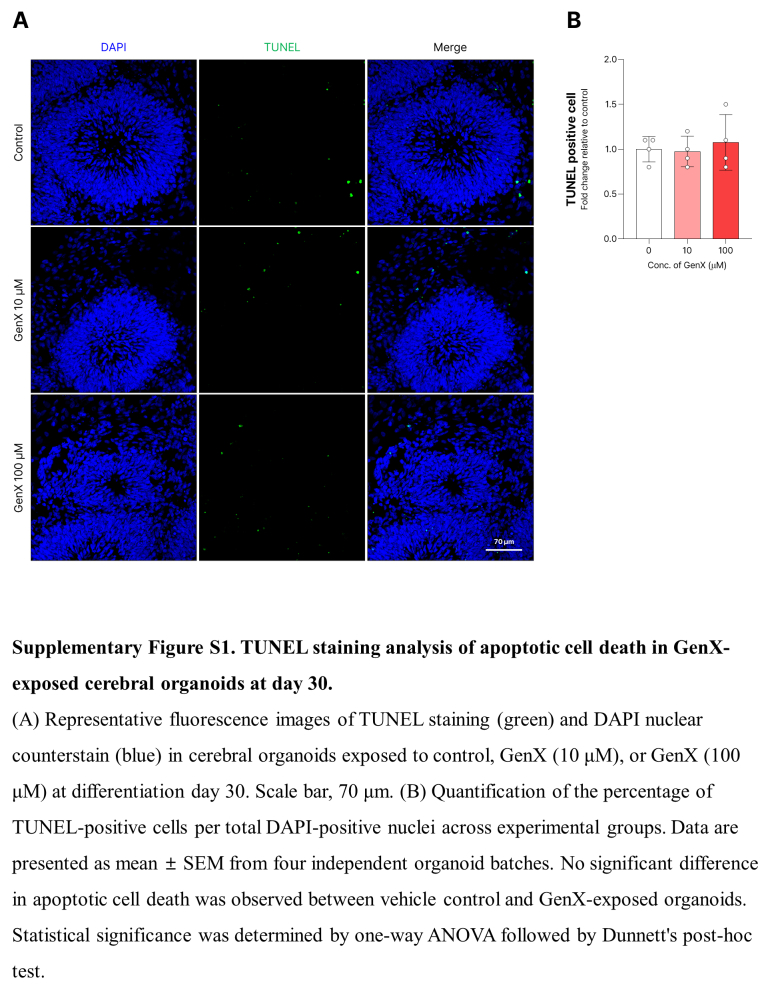


**figs2:**
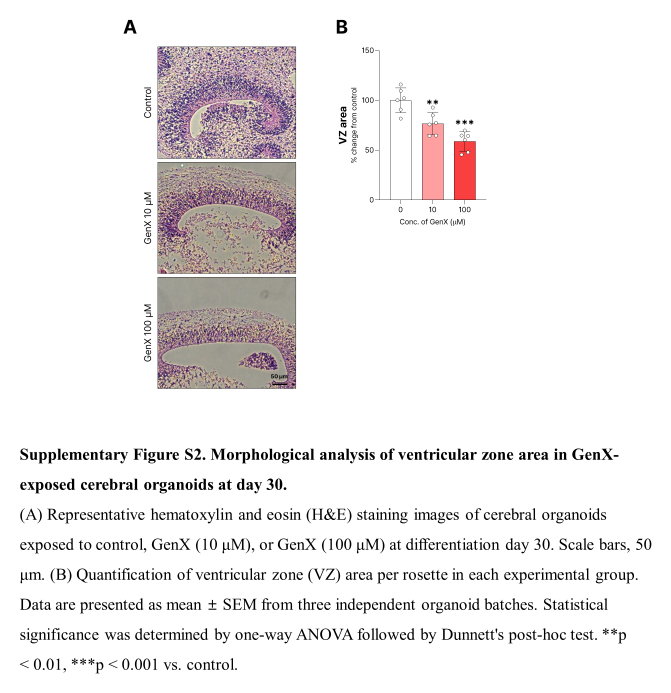


**figs3:**
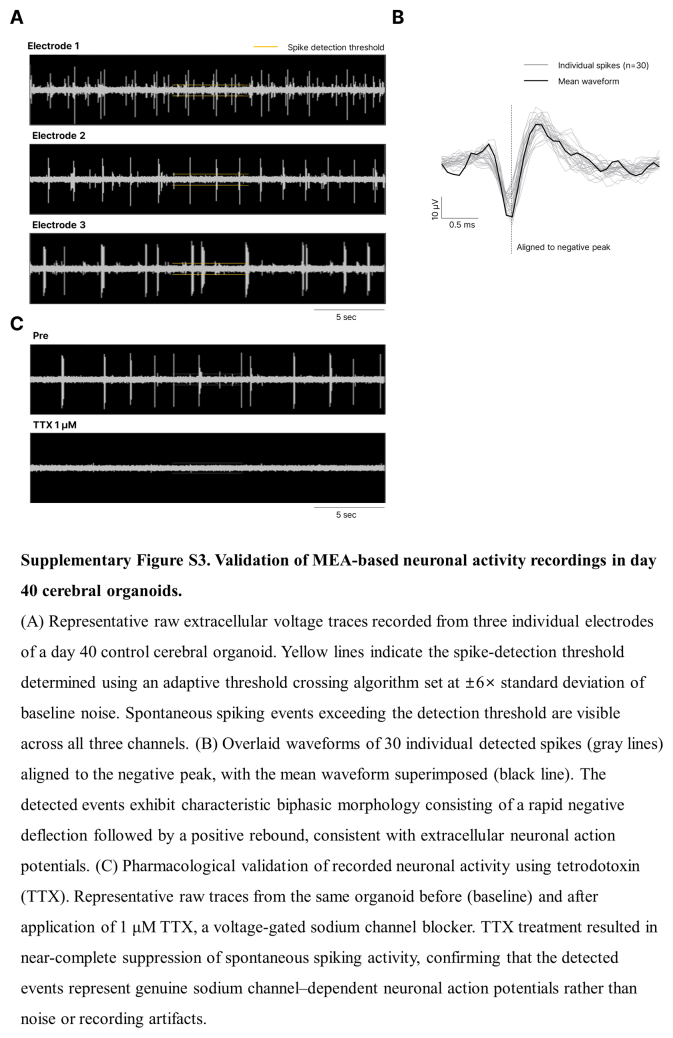


## Data Availability

Data will be made available on request.
